# The diagnostic value of multimodal imaging for myocardial injury in heat stroke

**DOI:** 10.3389/fcvm.2026.1741789

**Published:** 2026-04-13

**Authors:** Guoxiu Lu, Jingjing Liang, Qi Peng, Shanhu Hao, Guoxu Zhang

**Affiliations:** Department of Nuclear Medicine, General Hospital of Northern Theatre Command, Shenyang, China

**Keywords:** echocardiography, heat stroke, magnetic resonance imaging, multimodal imaging, myocardial injury, myocardial perfusion imaging, prognosis

## Abstract

**Objective:**

This study aimed to evaluate and compare the diagnostic and prognostic value of a multimodal imaging approach—integrating echocardiography (US), cardiac magnetic resonance imaging (CMRI), and myocardial perfusion imaging (MPI)—for detecting myocardial injury (MI) secondary to heat stroke (HS).

**Methods:**

This single-center retrospective study analyzed data from 187 HS patients (49 with MI, 138 without MI) and 20 healthy controls (HC). The diagnostic accuracy of echocardiography (US), cardiac magnetic resonance (CMR), and MPI, individually and in combination, was evaluated against a composite clinical reference standard. Receiver operating characteristic curve (ROC) compared the HS patients with the MI group (positive cases) against a combined negative group comprising all HS without MI patients and HCs. The association between imaging parameters and a 30-day composite clinical endpoint (all-cause death, heart failure, or significant arrhythmia) was analyzed using logistic regression.

**Results:**

Among single modalities, MPI demonstrated the highest diagnostic efficacy (area under the curve, AUC = 0.788), followed by CMRI (AUC = 0.721) and US (AUC = 0.648). The combination of all three modalities (US + CMRI + MPI) achieved the highest diagnostic performance (AUC = 0.861, sensitivity 85.7%, specificity 87.3%, accuracy 87.0%), significantly outperforming any single modality or dual-modality combination (*P* < 0.01). Multivariate analysis identified the extent of myocardial necrosis (assessed by CMRI-derived extracellular volume, extracellular volume; Odds Ratio, OR = 5.2, *P* = 0.003) and the severity of myocardial ischemia (assessed by MPI summed stress score, SSS; OR = 3.8, *P* = 0.017) as independent predictors of adverse 30-day outcomes.

**Conclusion:**

A multimodal imaging strategy combining US, CMRI, and MPI provides superior diagnostic accuracy for detecting HS-induced myocardial injury compared with any single modality alone. Furthermore, it offers significant prognostic value by identifying the extent of myocardial necrosis and severity of ischemia, which serve as independent risk factors for short-term adverse events. Thus, this approach may facilitate risk stratification and inform clinical decision-making and patient management.

## 1 Introduction

Heat stroke (HS) is a life-threatening medical emergency. It is characterized by a profound thermoregulatory dysfunction following exposure to extreme environmental heat or strenuous physical exertion. It culminates in a precipitous rise in core body temperature (>40°C), leading to multiorgan dysfunction syndrome (1). HS is classified into classic heat stroke (CHS) and exertional heat stroke (EHS) based on the causes of the disease and susceptible populations. Its features include subcutaneous vasodilation, splanchnic ischemia, arterial hypotension, intracranial hypertension, and decreased cerebral perfusion, resulting in the failure of multiple organs (including the heart and brain) (2). Among the severe complications of heat stroke, acute myocardial injury (MI) is increasingly recognized as a critical determinant of patient outcomes (3, 4). Previous studies have demonstrated that HS patients (5, 6) and rodents (7, 8) exhibited arterial hypotension and myocardial infarction. Oxidative stress and inflammation contribute to the genesis of HS-induced myocardial and brain damage (9, 10).

Heat exposure increases the risk of cardiovascular mortality, with approximately 43.4%–74.6% of HS patients exhibiting cardiovascular dysfunction (11, 12). A meta-analysis of patients with HS showed that pre-existing cardiovascular disease induced a significant 2.5-fold increase in risk of mortality (13). Cardiovascular abnormalities often manifest early in HS and are characterized by heart failure, focal myocardial necrosis, and arrhythmias (1). Survivors of HS may develop long-term cardiovascular sequelae (14). These findings highlight the profound impact of cardiac injury on the prognosis of patients with HS and the underscore the need for focused studies on therapeutic or preventive strategies for HS-induced myocardial injury.

Early and accurate diagnosis of HS-induced myocardial injury remains a significant clinical challenge. Xiang (15) proposed c-Jun as a novel biomarker for early diagnosis and prognostic evaluation of HS-induced myocardial injury. However, the predictive model was developed using a limited clinical sample size of single-center origin, emphasizing the need for validation using a large sample size of multicenter origin. Current diagnostic approaches primarily depend on clinical presentation and laboratory biomarkers, which, while necessary, lack specificity. Elevated biomarkers such as creatine kinase (CK) and troponin may originate from skeletal muscle rhabdomyolysis—a common feature of heat stroke. Chao (16) reported that rats with EHS onset displayed skeletal muscle damage, elevated serum levels of skeletal muscle damage indicators (e.g., creatinine kinase, myoglobin, and potassium), and myocardial injury indicators (e.g., cardiac troponin I, creatinine kinase, and lactate dehydrogenase), returning to homeostasis within 3 days following EHS. However, EHS-induced myocardial damage, pathological echocardiography, myocardial fibrosis, hypertrophy, and deposited misfolded proteins lasted up to 14 days following EHS at least. This complicated the cardiac-specific interpretation of the results, potentially leading to misdiagnosis or delayed recognition of true cardiac involvement (17, 18).

Recently, the demand for more accurate and precise diagnostic tools has become increasingly apparent. Multimodal cardiovascular imaging provides a robust approach by directly visualizing and characterizing myocardial tissue characteristics. Echocardiography (US) provides real-time assessment of cardiac structure and function (19). Cardiac magnetic resonance (CMR) imaging enables high-resolution tissue characterization and the detection of edema, necrosis, and fibrosis (20). Zhang (21) used CMR-derived strain analysis to predict return to training after exertional heat stroke. They further reported that two-dimensional global longitudinal strain (≤−15.0%) serves as an incremental prognostic CMR biomarker for predicting return to training in soldiers with EHS. Myocardial perfusion imaging (MPI) evaluates regional blood flow and viability (22). While each modality provides unique insights, their integrated application has shown superior diagnostic and prognostic value in various cardiomyopathies (23). However, the systematic evaluation of a combined multimodal imaging strategy specifically for heat stroke-induced myocardial injury remains limited.

This study aims to address this gap. We comprehensively evaluate and compare the diagnostic efficacy of US, CMR, and MPI, both individually and in combination, for detecting myocardial injury in heat stroke patients. Furthermore, we explore the utility of these imaging techniques in assessing the severity of cardiac damage and their correlation with short-term clinical prognosis.

## 2 Methods

### 2.1 Study population

This single-center, retrospective study analyzed the clinical and imaging data of 305 patients with confirmed heat stroke who were admitted to our hospital between June 2010 and January 2025. For the HS group, patients were eligible if they met the following criteria: (1) clinical diagnosis of heat stroke: based on ICD-10 criteria (T67.0) (24), including hyperthermia (>40 °C), central nervous system dysfunction, and exposure to heat stress (25); (2) age ≥18 years; (3) availability of complete laboratory examination data; and (4) completion of US, CMR, and MPI once hemodynamically stable [median time: 18 h post-admission; interquartile range (IQR): 12–24 h]. The exclusion criteria were as follows: (1) history of structural heart disease, coronary artery disease, hypertension, diabetes, or other serious systemic diseases; (2) pregnancy or lactation; (3) poor quality imaging or laboratory data; and (4) inability to accurately assess laboratory results.

For the HS participants with MI, besides the above-mentioned four inclusion criteria, they were confirmed using a pre-specified composite clinical reference standard that integrated multiple data sources to minimize incorporation bias: (1) biochemical evidence: elevated high-sensitivity troponin T (hs-TnT ≥ 14 ng/L) during hospitalization, with exclusion of myocardial infarction, pulmonary embolism, severe infection, and renal insufficiency; (2) presence of abnormal heart symptoms (including ischemic heart disease, stroke, heart failure, or arrhythmias), with alternative causes excluded by coronary angiography or computed tomography coronary angiography performed during the index admission (26), thus confirming the absence of obstructive coronary artery disease;and (3) clinical course consistency: a temporal association between the heat stroke event and biomarker elevation, with subsequent biomarker dynamics consistent with myocardial injury. Notably, imaging findings from the index tests (US, MRI, or MPI) were not included in this reference standard to avoid incorporation bias.

**Figure 1:**
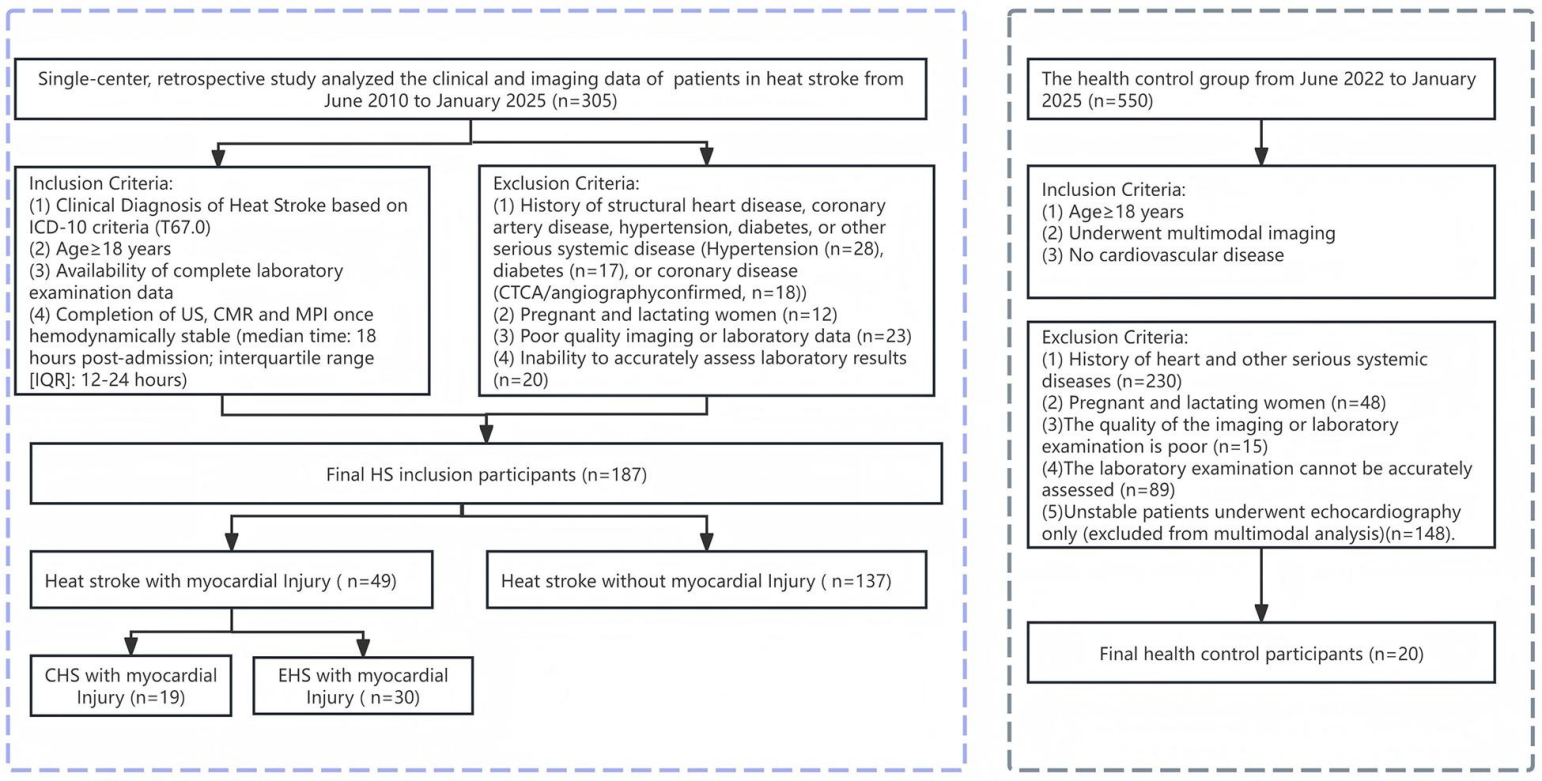
Study population flowchart. The flowchart shows the selection process, HS participants with MI, HS participants without MI, and healthy control based on eligibility. HS, heat stroke; MI, myocardial injury; CHS, classic heat stroke; EHS, exertional heat stroke.

A control group of 20 healthy volunteers was retrospectively selected from our institutional database. All controls had undergone the same multimodal imaging protocol for evaluation of non-specific symptoms (e.g., atypical chest pain). They were subsequently confirmed to have no cardiovascular disease. All had normal cardiac biomarkers, electrocardiogram (ECG), and imaging results. A flowchart of participant selection for HS with MI, HS without MI, and controls is shown in Figure 1.

### 2.2 Blood sampling

Blood samples were collected immediately before imaging examinations for all participants. The following blood sampling parameters were measured within 24 h of symptom onset: serum CK, creatine kinase-MB isoenzyme (CK-MB), myoglobin (Myo), N-terminal pro-B-type natriuretic peptide (NT-proBNP), amylase (AMY), C-reactive protein (CRP), and high-sensitivity troponin T (hs-TnT), in addition to routine blood tests. All blood samples were processed at our hospital laboratory using standardized commercially available test kits.

### 2.3 Imaging examination methods

#### 2.3.1 Ultrasound

US was performed using a Philips iE33 ultrasound system equipped with a 1–5 MHz transducer. Standard layers and apical views were obtained. The following parameters were recorded: left ventricular end-diastolic diameter (LVEDD), left ventricular end-systolic diameter (LVESD), wall motion score index (WMSI), left ventricular ejection fraction (LVEF), stroke volume (SV), and cardiac output (CO). Strain analysis was not part of the routine study protocol and is therefore not reported.

#### 2.3.2 Cardiac magnetic resonance

CMR was performed on a GE Discovery 750 3.0T scanner using a dedicated cardiac coil. The protocol included: (1) localizers; (2) T2-weighted imaging for edema assessment; (3) cine imaging for functional assessment; (4) first-pass perfusion; (5) late gadolinium enhancement (LGE) imaging. Quantitative T2 and extracellular volume (ECV) analyses were performed using post-processing software.

#### 2.3.3 Myocardial perfusion imaging

Resting MPI was performed using 99mTc-sestamibi (740 MBq). Images were acquired 60 min following injection using a SPECT/CT system (Siemens Symbia T2). Images were reconstructed and analyzed quantitatively using the Corridor 4DM software package (INVIA, Ann Arbor, MI, USA). Perfusion defects were quantified using the 17-segment model. A summed stress score (SSS) >3 was considered abnormal (27).

### 2.4 Image analysis and diagnostic criteria

All images were analyzed independently by two experienced physicians who were blinded to clinical data and other imaging results. Positive imaging findings for myocardial injury were defined as follows: (1) US: LVEF <50% or regional wall motion abnormality in ≥2 segments (16, 21); (2) CMR: T2 mapping value ≥50 ms and/or LGE involving ≥5% of left ventricular mass or ECV > 28.0% (28); and (3) MPI: perfusion defect (≤50% tracer uptake) in ≥1 segment on the 17-segment mode or SSS scores >3 (29). Inter-observer agreement was excellent, with Cohen's kappa (*κ*) values of 0.78 for US, 0.82 for CMR, and 0.78 for MPI.

### 2.5 Statistical analysis

Data analysis was performed using SPSS 22.0 software. Continuous variables were expressed as mean ± standard deviation and compared using the t-test or Mann–Whitney *U*-test as appropriate. Categorical variables were expressed as rates and compared using the chi-square test. To evaluate the diagnostic efficacy of different imaging modalities, receiver operating characteristic (ROC) curve analysis was performed using the composite clinical reference standard as the gold standard. For the primary analysis of diagnostic test accuracy, the “positive” group was defined as HS patients with MI (*n* = 49). The “negative” group for ROC curve construction was defined as all participants without confirmed MI, encompassing both HS patients without MI (*n* = 138) and healthy controls (*n* = 20). The area under the curve (AUC), sensitivity, specificity, and accuracy were calculated. Prognostic analysis employed univariate and multivariate logistic regression, with a 30-day composite endpoint (all-cause death, heart failure, or significant arrhythmia) as the dependent variable. A *P* < 0.05 was considered statistically significant.

## 3 Results

### 3.1 Baseline characteristics

A total of 190 hospitalized participants were enrolled in the baseline multimodal study. After exclusion of participants without contact information (*n* = 1) and participants who refused to participate in the follow-up (*n* = 2), a total of 187 HS participants were included in the final analysis. Of these, 138 had HS without myocardial injury (median age, 40.65 years; IQR, 20–53 years) and 49 had HS with myocardial injury (median age, 40.58 years; IQR, 19–64 years). A healthy control group (*n* = 20) with participants of a similar training level and age distribution (median age, 38.0 years; IQR, 19–50 years) were also enrolled. As shown in Table 1, at baseline upon admission (within 24 h of onset), laboratory indicators reflecting myocardial injury (CK, CK-MB, NT-ProBNP, CRP, hs-TnT) were significantly elevated in HS with MI participants, while no significant differences were observed between the HS without MI participants and healthy controls.

**Table 1:** Participants' baseline information.

Characteristics	HS with MI	HS without MI	Health control	Value	*P*-value
	(*n* = 49)	(*n* = 137)	(*n* = 20)		
Age (years)	40.58 (19.64)	40.65 (20.53)	38.00 (19.50)	2.516	0.083
Male, *n* (%)	39 (79.6%)	112 (81.2%)	16（80.0%）	0.063	0.969
BMI (kg/m^2^)	22.02 ± 0.54	22.13 ± 0.53	21.93 ± 0.65	1.228	0.295
Symptoms					
Chest pain, *n* (%)	30 (61.2%)	5 (3.6%)	2 (10.0%)	82.666	<0.001
Exertional dyspnea *n*, (%)	27 (55.1%)	8 (5.8%)	1 (5.0%)	63.553	<0.001
Syncope, *n* (%)	32 (65.3%)	4 (2.9%)	3 (15.0%)	92.317	<0.001
Dizziness, *n* (%)	35 (74.5%)	2 (1.4%)	1 (5.0%)	126.478	<0.001
Systolic BP (mmHg)	114.46 ± 8.99	114.27 ± 8.92	116.90 ± 9.61	1.017	0.311
Diastolic BP (mmHg)	84.14 ± 5.77	85.31 ± 6.09	85.20 ± 6.13	0.970	0.339
Heart rate (bpm)	107 (91,125)	83 (59,104)	80 (60,95)	11.665	<0.001
ECG abnormal, *n* (%)	46 (93.9%)	10 (7.2%)	0 (0.0%)	147.365	<0.001
CK (U/L)	1,111.63 ± 61.83	445.33 ± 17.45	107.75 ± 615	64.728	<0.001
CK-MB (U/L)]	55.71 ± 2.23	20.27 ± 0.53	11.30 ± 0.44	57.199	<0.001
Myo (ng/mL)	720.80 ± 50.38	244.80 ± 11.89	44.65 ± 4.54	70.859	<0.001
NT-ProBNP (pg/mL)	824.69 ± 38.14	179.33 ± 5.82	76.95 ± 9.17	110.228	<0.001
AMY (U/L)	52.30 ± 4.03	54.61 ± 2.31	66.60 ± 5.24	1.566	0.211
CRP (mg/L)	44.43 ± 1.93	3.01 ± 0.35	4.45 ± 0.49	97.521	<0.001
HS-TNT (ng/L)	7.94 ± 1.09	4.24 ± 0.18	2.95 ± 0.30	87.231	<0.001

HS, heat stroke; MI, myocardial Injury; BMI, body mass index; ECG, electrocardiogram; CK, creatine kinase; CK-MB, creatine kinase-MB isoenzyme; Myo, myoglobin; NT-pro BNP, N-terminal pro-B-type natriuretic peptide; AMY, amylase; CRP, C-reactive protein; hs-cTnT, high-sensitivity troponin T high-sensitivity cardiac troponin T.

Among the 49 HS with MI participants, dizziness (74.5%, 35/49) and syncope were the most common cardiac symptoms (65.3%, 32/49), followed by chest pain (61.2%, 30/49) and exertional dyspnea (55.1%, 27/49). In addition, two participants (4.1%, 2/49) suffered from recurrent heat illness after discharge. These abnormal cardiac symptoms occurred at a significantly higher frequency in the HS with MI group than in the HS without MI and healthy control groups.

**Figure 2:**
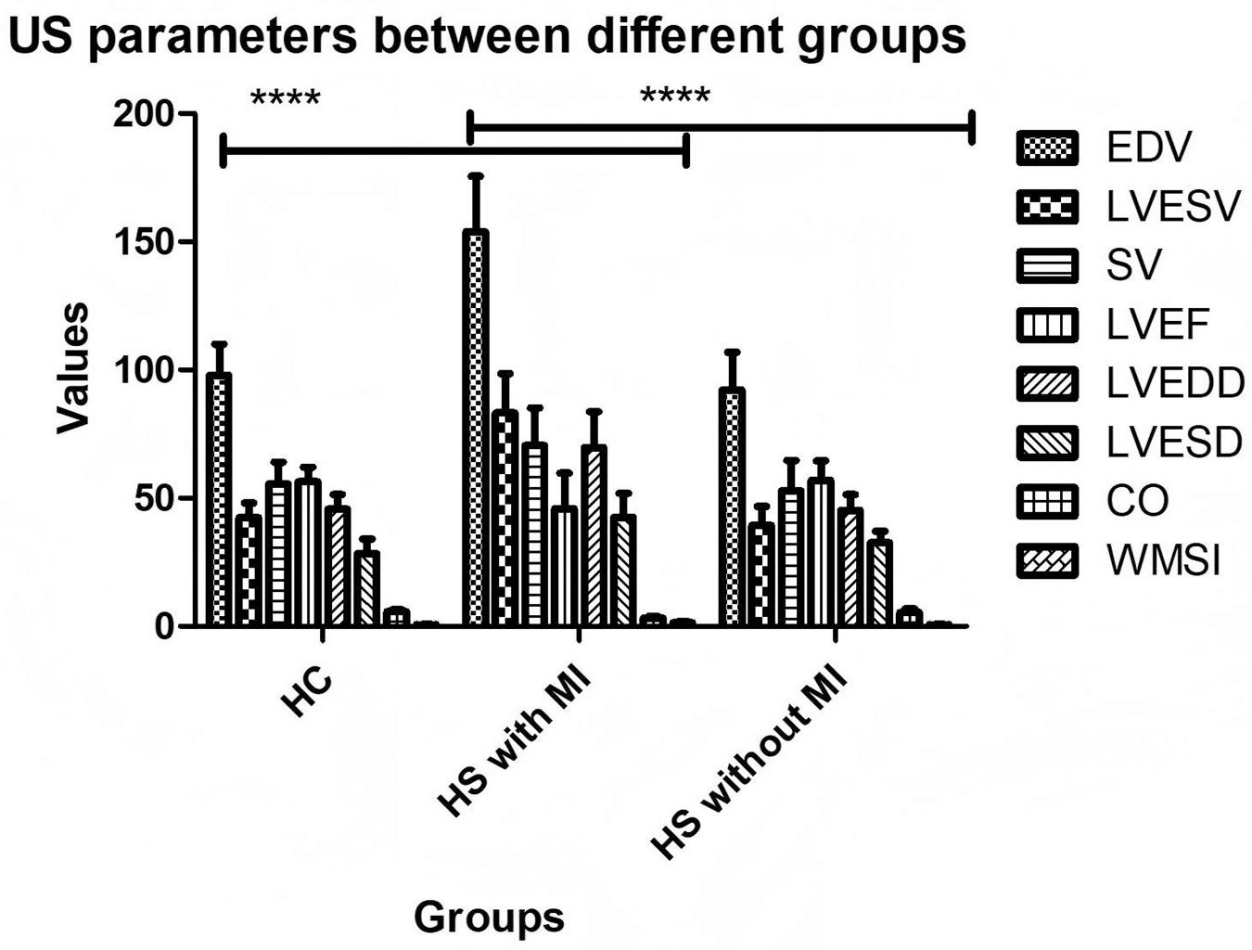
The parameters in US imaging of participants.

Figure 2 compares echocardiographic (US) parameters among three groups: healthy controls, heat stroke (HS) patients with myocardial injury (MI), and HS patients without MI. Relative to healthy controls, the HS with MI group showed significantly increased LVEDD, LVESD, LVESV, and SV, as well as significantly decreased CO and LVEF. WMSI was also significantly higher in the HS with MI group (*P* < 0.001), indicating distinct regional wall motion abnormalities. The HS without MI group showed no such significant abnormalities in ultrasound measurements compared with healthy controls.

Among the 49 HS participants with myocardial injury, 19 were cases of classic heat stroke (CHS) and 30 were exertional heat stroke (EHS). Both CHS and EHS subgroups demonstrated significantly elevated LVEDD, LVESD, LVESV, and SV, accompanied by significantly reduced CO and LVEF. WMSI was significantly higher in both patient subgroups than in the healthy control group (*P* < 0.001), confirming the presence of wall motion abnormalities. No significant differences in any ultrasound parameter were observed between the CHS and EHS subgroups (*P* > 0.05).

Among the 49 HS participants with myocardial injury, there were 19 cases of CHS and 30 cases of EHS. Both the CHS and EHS subgroups showed significantly increased LVEDD, LVESD, LVESV, and SV, along with significantly decreased CO and LVEF. WMSI was also significantly higher in the patient groups compared with the control group (*P* < 0.001), indicating the presence of wall motion abnormalities. No significant differences in US parameters were observed between the CHS and EHS groups (*P* > 0.05).

With regard to the CMR imaging, T2 values were elevated in 17 patients—15 patients in the HS with MI group and one patient each in HS without MI group and the healthy control group. Gadolinium contrast enhancement revealed subepicardial or medial linear enhancement in 13 patients; 10 patients (20.4%,10/49) showed positive enhancement in the HS with MI group, while three patients showed false positive signs in the HS without MI group. ECV values increased in the HS with MI groups but did not differ between the HS without MI and healthy control groups.

In terms of the MPI imaging, in the HS with MI group, 32 patients (65.3%, 32/49) presented with myocardial perfusion defects. The number of abnormal segments was 5.08 ± 1.94 and the SSS score was 15.50 ± 4.28. Only four patients (2.9%, 4/138) showed myocardial perfusion defects in the HS without MI group and one patient (5%,1/20) showed myocardial perfusion abnormalities due to high heart rate.

### 3.2 Comparison of imaging diagnostic efficacy

**Figure 3:**
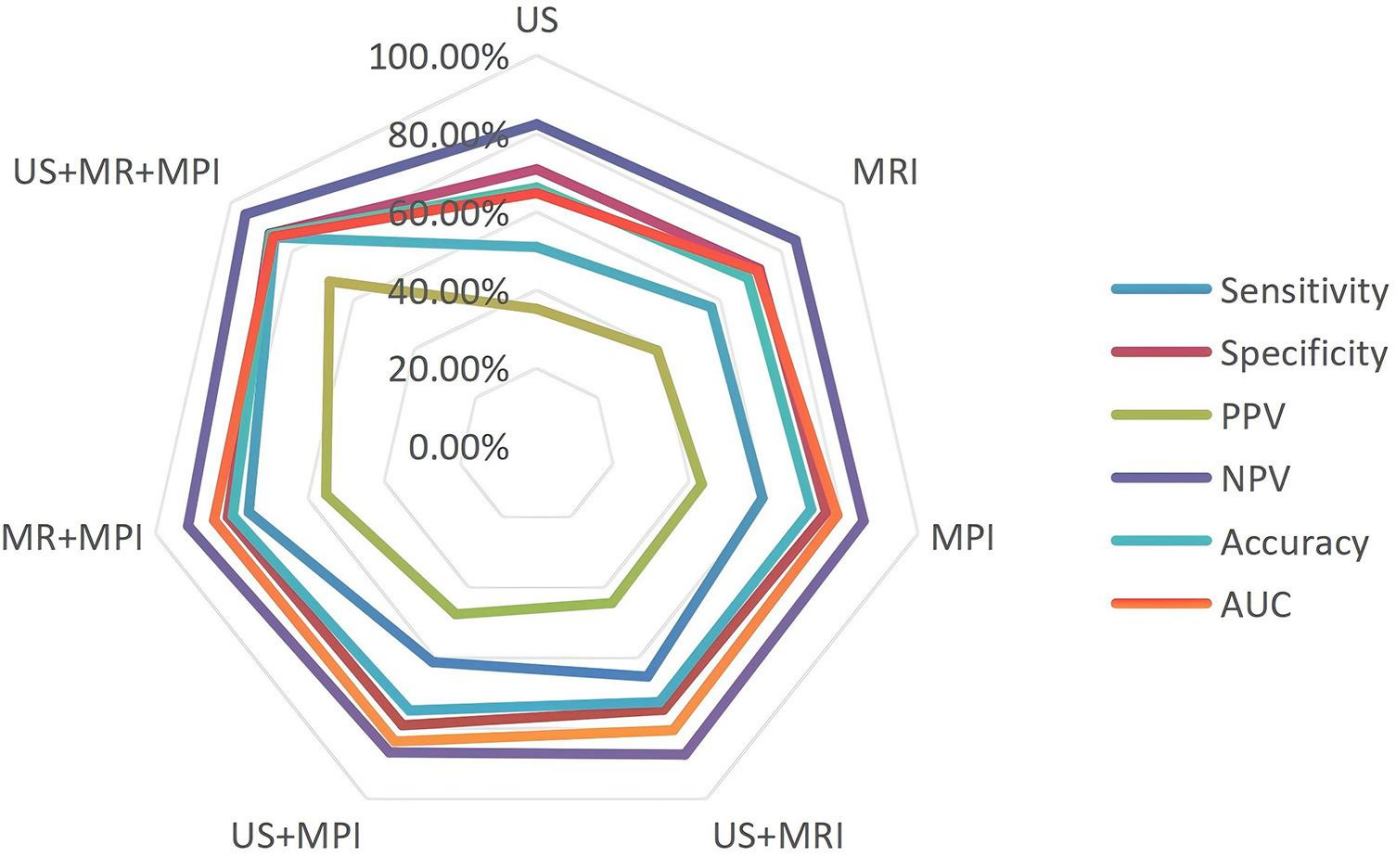
Comparison of diagnostic efficacy of different imaging methods for heat stroke-induced myocardial injury. PPV, positive predictive value; NPV, negative predictive value; AUC, the area under the curve.

Using the composite clinical reference standard as the gold standard, diagnostic thresholds were fixed *a priori* based on clinical standards and applied consistently to all participants. The diagnostic performance of each imaging modality and their combinations is presented in Table 2 and illustrated in Figure 3. ROC and corresponding AUC values were recalculated based on the final cohort, which compared HS patients with MI (*n* = 49) against the combined negative group of all participants without MI (*n* = 158). Among the single modalities, MPI demonstrated the highest diagnostic efficacy with an AUC of 0.788 (95% CI: 0.721–0.850). The AUCs for US and CMR were 0.648 (95% CI: 0.567–0.726) and 0.721 (95% CI: 0.645–0.793), respectively.

**Table 2:** The diagnostic performance of imaging modality and their combinations.

Imaging	Sensitivity	Specificity	PPV (%)	NPV (%)	Accuracy (%)	AUC (95% CI)
US	51.0% (36.5%–64.5%）	70.9% (63.2%–77.7%）	35.2	82.4	66.2	0.648 (0.567–0.726)
MRI	57.1% (42.2%–71.2%)	72.8% (65.2%–79.4%）	39.4	84.6	69.1	0.721 (0.645–0.793)
MPI	59.2% (44.3%–72.9%)	75.9% (68.6%–82.3%）	43.3	85.7	72.0	0.788 (0.721–0.850)
US + MRI	65.3% (50.5%–78.2%)	74.7% (67.2%–81.2%）	44.4	87.4	72.5	0.805 (0.741–0.862)
US + MPI	61.2% (46.3%–74.8%)	79.1% (72.0%–85.1%）	47.6	86.8	74.9	0.837 (0.778–0.889)
MR + MPI	75.5% (61.2%–86.7%)	81.0% (74.1%–86.8%）	55.2	91.4	79.7	0.846 (0.789–0.896)
US + MR + MPI	85.7% (72.8%–93.9%)	87.3% (81.2%–92.0%）	67.7	95.2	87.0	0.861 (0.807–0.907)

PPV, positive predictive value; NPV, negative predictive value; AUC, the area under the curve.

Multimodal combinations significantly improved diagnostic performance. The US + CMR + MPI combination achieved the highest AUC of 0.861 (95% CI: 0.807–0.907), with a sensitivity of 85.7% and a specificity of 87.3%. Notably, even within this stringent analytical framework, the tri-modal imaging combination (US + MRI + MPI) maintained a balanced diagnostic performance. The tri-modal combination also correctly identified most subjects in the negative group, with a specificity of 87.3%, whereas each single modality yielded more false-positive results. The optimal diagnostic cutoff values were LVEF≤48.5% for US; SSS score≥10.5 for MPI; and ECV≥28.5% for CMR. This underscores the potential clinical utility of multimodal imaging for detecting myocardial injury in heat stroke patients.

### 3.3 Association between imaging findings and clinical prognosis

During a median follow-up of 30 days, among the 49 HS with MI patients, 35 (71.4%) achieved complete recovery, 8 (16.3%) had persistent organ dysfunction, and 6 (12.2%) patients died. In contrast, among the 138 HS without MI patients, 128 (86.9%) completely recovered, while 12 (8.7%) died due to central nervous system failure (5, 5.2%), renal failure (2, 1.4%), and coagulation dysfunction (3, 2.1%).

Univariate analysis indicated ECV (assessed by MRI), SSS scores (assessed by MPI), initial LVEF (assessed by US), and age as significantly associated with adverse outcomes (*P* < 0.05). After incorporating these factors into a multivariate logistic regression analysis, the results showed that the ECV (OR = 5.2, 95% CI: 1.8–15.1; *P* = 0.003) and SSS scores (OR = 3.8, 95% CI: 1.3–11.2; *P* = 0.017) were independent risk factors affecting patient 30-day prognosis. For the 180-day follow-up, we found that the SSS scores (OR = 2.3, 95% CI: 1.7–9.5; *P* = 0.032) alone were the independent risk factors.

## 4 Discussion

Cardiac imaging is an essential step in the initial evaluation of patients with suspected heat stroke, serving both diagnostic and prognostic purposes (30). Myocardial injury in HS is a critical complication driven by systemic inflammation (31), oxidative stress (32), and direct thermal damage (24). Early identification of abnormal cardiac abnormalities in heat stroke helps guide targeted treatments, forecast adverse outcomes, and direct further clinical evaluation and monitoring. This study is the first to systematically evaluate the diagnostic and prognostic value of multimodal imaging (US, CMR, and MPI) for heat stroke-induced myocardial injury. The main findings of this study are as follows: First, among single imaging modalities, MPI demonstrated the highest diagnostic efficacy (AUC 0.788). Second, the combination of all three modalities significantly enhanced diagnostic accuracy, with an AUC of 0.861. Finally, the extent of myocardial necrosis assessed by MRI-derived ECV and the severity of ischemia assessed by MPI-derived SSS were independent predictors of 30-day adverse outcomes.

The mechanism of myocardial injury in heat stroke is complex, involving systemic inflammation, oxidative stress, direct thermal damage, and potential microcirculatory dysfunction (33–35). High temperatures may be associated with increased hospital encounters for total acute cardiovascular disease, hyper/hypotension, acute myocardial infarction, and ischemic stroke (36–39). However, few studies have reported on cardiovascular mortality in HS patients with MI, and findings have been inconsistent. This inconsistency may result from different standards and definitions across different studies. Given its versatility and availability, US is the first-line imaging modality for characterizing new cardiomyopathies or monitoring clinical changes in heat stroke patients. Although cardiac MRI holds advantages in tissue characterization, the relatively low proportion of myocardial necrosis (LGE-positive) found in this acute-phase population may reflect injury in the early edema phase rather than a fibrosis stage (21, 28, 40). The relatively high diagnostic value of MPI observed in this study provided a hypothesis that warrants future investigation. It may reflect myocardial perfusion abnormalities due to coronary microcirculatory impairment or endothelial dysfunction under heat stress (41, 42). Therefore, in this study, we adopted the composite clinical reference standard as the gold standard. The application of laboratory indicators combined with US, CMR, and MPI multimodal imaging examination improved the accuracy of HS with MI diagnosis. Early, accurate diagnosis can improve outcomes for HS patients.

The findings of this study have implications for clinical practice. US, as a first-line screening tool, is convenient but may miss early or subtle injury. For high-risk patients (e.g., those with significantly elevated troponin, hemodynamic instability, or ECG abnormalities), early multimodal imaging assessment is needed, particularly combining MRI and MPI. The combination multimodal approach allows comprehensive evaluation of the nature and extent of myocardial injury accurately. HS patients with persistently and significantly elevated troponin, clinical symptoms or signs of heart failure or arrhythmia, or abnormal or inconclusive initial echocardiography should undergo proactive multimodal imaging evaluation for HS-related myocardial injury. This stratified approach can optimize test selection, particularly in resource-limited settings.

The strengths of this study include the first comprehensive comparison of three major cardiac imaging techniques, and the use of a diagnostic standard that avoided imaging self-referencing. However, we must acknowledge its limitations. First, the sample size was relatively small, and the single-center retrospective design may introduce selection bias. Second, the prognostic follow-up period was short, only 30 days, limiting assessment of long-term sequelae of heat stroke myocardial injury, such as chronic fibrotic remodeling and arrhythmic risk. Third, more sensitive strain imaging techniques were not included. Fourth, the heterogeneity of troponin elevation (potentially originating from myocardial injury, rhabdomyolysis, or systemic inflammation) remains a challenge, complicating our diagnostic interpretation.

Furthermore, the observed decrease in AUC values for all imaging strategies upon inclusion of HS patients without MI in the control group provides a more realistic estimate of their discriminative power in target clinical populations. The initially higher AUCs (e.g., 0.928 for the tri-modal combination) represented performance against an “ideal” negative group (healthy controls), which may lead to an overestimation of specificity compared to a real-world clinical scenario. On the contrary, the final reported AUCs (e.g., 0.861) reflect the challenge of distinguishing myocardial injury within a cohort where all patients have the primary condition of heat stroke. This adjustment is crucial for clinical translation, as differential diagnosis must occur among sick patients. Future studies should compare imaging findings in HS patients with versus without MI to establish true clinical discriminated value. Large-scale, prospective, long-term follow-up studies are needed to validate these findings. In addition, the application of artificial intelligence-assisted multimodal radiomics may enhance precision in diagnosis and prognosis prediction.

## 5 Conclusion

Multimodal imaging techniques (US, CMR, and MPI) hold significant value in the diagnosis and management of heat stroke-induced myocardial injury. The multimodal combination (US + MRI + MPI) provides the highest diagnostic efficacy. Among single modalities, MPI demonstrated the best diagnostic performance in this study population, not MRI. The extent of myocardial necrosis assessed by MRI and the severity of ischemia assessed by MPI were independent predictors of short-term prognosis. Although challenges in cost and standardization persist, multimodal imaging represents a new paradigm for evaluating heat stroke-induced myocardial injury, providing a basis for risk stratification and the development of individualized clinical management plans. Future research should focus on longitudinal studies to validate multimodal biomarkers and optimize therapeutic outcomes.

## Data Availability

The original contributions presented in the study are included in the article/Supplementary Material, further inquiries can be directed to the corresponding authors.
